# mTOR在肺类癌中的研究进展

**DOI:** 10.3779/j.issn.1009-3419.2013.01.08

**Published:** 2013-01-20

**Affiliations:** 100730 北京，中国医学科学院北京协和医学院 北京协和医院呼吸内科 Department of Respiratory Diseases, Peking Union Medical college Hospital, Peking Union Medical College and Chinese Academy of Medical Sciences, Beijing 100730, China

**Keywords:** 肺类癌, 雷帕霉素靶蛋白, 药物治疗, Lung carcinoid tumors, Mammalian target of rapamycin, Medical treatment

## Abstract

雷帕霉素靶蛋白（mammalian target of rapamycin, mTOR）作为PI3K/AKT/mTOR路径中主要的蛋白激酶，在胞内调节细胞生长、分化、凋亡以及肿瘤血管生成的信号传导中起重要作用。近年来人们发现mTOR及其相关激酶在神经内分泌肿瘤中表达升高，于是特异性降低mTOR表达的药物成为继手术治疗肺类癌之外的又一选择。

## Abstract (en)

Mammalian target of rapamycin (mTOR), a main protein kinase in the phosphoinositide 3-kinase (PI3K)/AKT/mTOR signaling pathway, is an important intracellular mediator involved in multiple celluar functions including proliferation, differentiation, apoptosis, tumorigenesis, and angiogenesis. Recently, the high expression of mTOR and mTOR-related kinase have been found in neuroendocrin tumors. Therefore, mTOR pathway represents an attractive target for new anticancer therapies except surgery.

肺类癌是发生于肺和支气管上皮的神经内分泌肿瘤，每年的发病率约为1.35/100, 000人，占所有肺恶性肿瘤的1%-2%。近年来，肺类癌的发病率逐渐上升，存活率与以前的文献相比也有升高，但平均发病年龄没有下降，确诊前依然有5年-7年的潜伏期，并且60%-70%的患者在确诊前通常已有转移^[1, 2]^。其中，19%-39%的患者是无症状的，5%-10%的患者由于肿瘤细胞产生的血清素等生物活性胺类物质被释放入血而表现为类癌综合征，其他患者可由于肿瘤本身局部物理作用而表现出刺激性咳嗽、痰中带血、胸痛、阻塞性肺炎等^[3, 4]^。传统上，手术切除是早期肺类癌患者的首选治疗方式。但是有类癌综合征或者晚期转移的患者还需要以铂类为基础的化学辅助治疗。随着时间的推移，包括干扰素、生长抑素类似物、雷帕霉素靶蛋白（mammalian target of rapamycin, mTOR）受体抑制剂在内的生物制剂应运而生。本文将围绕mTOR及其受体抑制剂在肺类癌方面的研究做一综述。

## 1 PI3K/AKT/mTOR路径

mTOR的活性变化主要通过PI3K/AKT/mTOR路径（图 1），其作为PI3K（phosphatidylinositol 3-kinase, PI3K）通路中的一个信号放大器，可以被胞外跨膜受体酪氨酸激酶类所激活，比如表皮生长因子受体（epidermal growth factor receptor, EGFR）、胰岛素样生长因子受体（insulin like growth factor 1 receptor, IGF-1R）、血管内皮生长因子受体（vascular endothelial growth factor receptor, VEGFR）等。另外，其它可以激活mTOR复合物的分子有肿瘤抑制因子磷酸酶和张力蛋白同源物（phosphatase and tensin homolog, PTEN）、多发性神经纤维瘤一型（neurofibromatosis type 1, NF1）、肝激酶B1（liver kinase B1, LKB1）、大鼠肉瘤（rat sarcoma, RAS）、迅速加速性纤维肉瘤（rapidly accelerated fibrosarcoma, RAF）以及细胞的能量、营养变化情况和应激状态。

**1:**
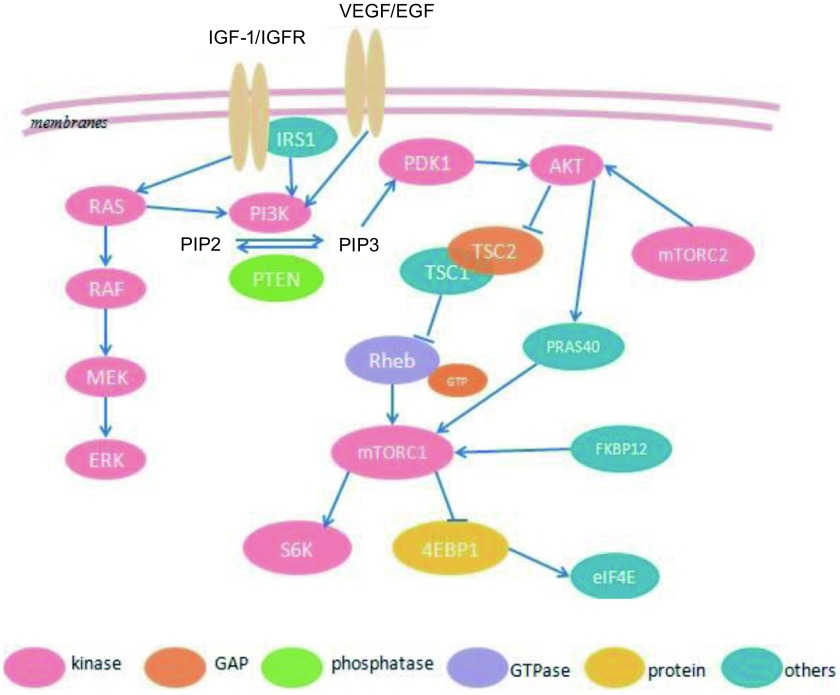
mTOR信号通路 mTOR signaling pathway

当细胞膜外的受体接受相应配体刺激后，可以激活PI3K/AKT/mTOR路径的上游因子。如果PI3K被活化，它可以通过招募并活化血浆细胞锚定蛋白受体使磷脂酰肌醇4，5二磷酸（phosphorylation of phospha-tidylinositol 4, 5-bisphosphate, PIP2）磷酸化为磷脂酰肌醇三磷酸（phosphatidylinositol 3, 4, 5-triphosphate, PIP3）。如果*PTEN*基因被激活，PIP3则去磷酸化为PIP2，从而抑制整个mTOR通路的活性。所以PTEN的缺失^[5]^、沉默或者突变会造成其功能的丧失，导致AKT或者整个mTOR路径的持续活化^[6]^。在丝/苏氨酸蛋白激酶（Serine/Threonine kinase, AKT）同PIP3的PH区域（血小板-白细胞c激酶底物类似物）结合后，可以通过抑制结节性硬化复合物2（tuberous sclerosis complex 2, TSC2）的活性，或者直接磷酸化PRAS40（mTORC1的一个组成部分）而激活mTORC1的活性。TSC2包含一个GTP酶激活区域，这个区域可以使Ras同源性GTP酶（Ras homolog enriched in brain GTP kinase, Rheb GTPase）沉默，而Rheb GTPase是mTOR的激活剂。所以TSC1/2复合物活性受抑制之后可以转而激活mTORC1。另外TSC2还可以由于AMP/ATP比值升高而被激活，所以体内能量减少可以引起AMP活化性蛋白激酶（AMP-activated protein kinase, AMPK）的活化，从而负性调节mTOR的活性^[7]^。

mTOR主要由mTOR复合物1（mTORC1）和mTOR复合物2（mTORC2）两部分组成，其中mTORC1起主要作用。当mTORC1被激活以后可以直接磷酸化真核生物起始因子4E结合蛋白1（4E binding protein 1, 4EBP1）和S6激酶（S6 kinase, S6K）。4EBP1作为一种帽结合蛋白，与真核生物起始因子4E（eIF4E）结合后可以激活转录过程。有文献^[8]^报道，对雷帕霉素耐药的细胞4EBP1表达水平降低，而S6K没有改变。在这些细胞里，对mTOR的抑制并不能改变帽依赖性的细胞转录。并且当这些细胞恢复了对雷帕霉素敏感性之后，4EBP1的水平也随之恢复。S6K被p-mTOR磷酸化成p70S6K后对肿瘤细胞所起的作用主要是减少细胞的大小。其不仅可以特异性的识别mRNA 5’端的帽子结构，影响mRNA的转录，还可以使胰岛素受体底物1（insulin receptor substrate 1, IRS-1）、真核生物转录起始因子4B（eukaryotic transcription initiation factor, eIF4B）、程序性细胞凋亡因子4（programmed cell death factor 4）、糖原合酶激酶3（glycogen synthase kinase 3）以及mTOR等磷酸化^[9]^。

与p70S6K对细胞的影响不同，PI3K、AKT、mTOR突变后不仅可以改变细胞的形态，还可以改变细胞的数量^[10]^。而在PTEN缺失的细胞系中，AKT会因此而持续活化，同时S6K1以及4EBP1磷酸化程度增加。而AKT持续活化的细胞在增殖上会变得更加依赖于mTOR路径的上调。这就使得这些*PTEN*基因阴性的细胞系或者动物移植模型对mTOR受体抑制剂的敏感性更高^[11]^。既往在肾癌中mTOR受体抑制剂的研究^[12]^表明，该抑制剂对mTOR的作用主要通过抑制下游S6K的活性，使细胞周期停滞在G_1_期，并且减少低氧诱导因子HIF的产生。也有研究^[7]^表明eIF4E以及S6K的高表达同肿瘤的不良预后有关。

## 2 mTOR在NSCLC中的研究

mTOR是哺乳动物胞内普遍存在的丝/苏氨酸蛋白激酶，异常活化的mTOR可以通过延迟细胞循环G_1_-S期而影响细胞的状态，从而导致多种肿瘤的发生发展。Valsamo等^[13]^研究了mTOR表达同患者特征的相关性后发现mTOR表达升高的非小细胞肺癌（non-small cell lung cancer, NSCLC）患者占总人数的50%，其中51.7%为腺癌患者。在此实验中收录的4例肺类癌患者mTOR表达均升高，而且mTOR高表达的患者比低表达的患者中位生存期长（52.7个月*vs* 38.5个月，*P*=0.06）。同时，该实验认为mTOR表达情况同患者组织分型、性别、年龄以及临床病理分期的关系无统计学意义。此实验说明了肺类癌是种高表达mTOR的肿瘤，并且mTOR表达率越高，患者生存期越长。与Valsamo等认为mTOR表达同患者临床病理分期无关相反，Mee-Hye等^[14]^发现临床病理分期较低的NSCLC患者更容易表达mTOR。而且Dhillon等^[15]^研究早期NSCLC患者中mTOR表达情况时，发现mTOR表达升高且没有淋巴结转移或者Ia期NSCLC患者预后不良（*P*值分别为0.016、0.001, 6）。并且，他们建议早期NSCLC患者（Ia期）可以考虑选择mTOR受体抑制剂进行治疗。

在研究了整体NSCLC中mTOR的表达情况之后，Luisella等^[16]^平行对比了24例肺典型类癌、73例肺不典型类癌、60例大细胞肺癌和61例小细胞肺癌的组织后发现，肺类癌中p-mTOR和p70S6K的表达明显高于大、小细胞肺癌（*P* < 0.001）。同时，在肺类癌患者中p-mTOR表达低的患者淋巴结转移率低（*P*=0.016），生存期延长（*P*=0.005）。另外，Johannes等^[17]^应用比较基因组杂交技术，分析了33例小细胞肺癌组织，13种小细胞肺癌细胞系，19例肺类癌以及9例胃肠道类癌的肿瘤组织。实验表明小细胞肺癌组织中PIK3CA（PI3K）、AKT1（akt）、PTEN（pten）以及FRAP1（mtor）的基因拷贝数明显高于肺类癌，分别为PIK3CA（75.8%-21.1%）、AKT（63.6%-26.3%）、PTEN（75.8%-21.1%）、FRAP1（54.5%-26.3%）。这个实验的结果同Luisella等使用Western blot以及免疫组化技术所得的结果截然相反。并且同所有肿瘤的平均发生率相比，肺类癌中只有AKT1（26.3% *vs* 16.3%）、FRAP1（26.3% *vs* 13.4%）表达高。

综上所述，我们可以发现mTOR在所有NSCLC中的表达情况以及同预后的关系至今没有定论。与NSCLC患者发病的分子机制相比[包括上游的酪氨酸激酶受体突变^[18, 19]^、PI3K/AKT/mTOR路径^[20, 21]^以及平行通路（RAS/RAF/MEK）的异常活化或基因突变]^[22, 23]^，肺类癌患者发病更多是由于mTOR的高表达。

## 3 mTOR受体抑制剂在肺类癌患者中的试验

mTOR抑制剂^[24, 25]^包括mTORC1抑制剂、mTORC1/mTORC2双重抑制剂、以及PI3K/mTOR双重抑制剂。mTORC1抑制剂包括坦西莫斯（Temsirolimus, CCI-779）、依维莫司（Everolimus, RAD001）以及雷帕霉素其它衍生物等; mTORC1/mTORC2双重抑制剂包括OSI-027;PI3K/mTOR双重抑制剂包括NVP-BEZ235、GDC-0980、GSK2126458、XL765等。根据分子量大小还可以分为大分子抑制剂和小分子抑制剂，其中大分子抑制剂有雷帕霉素（Rapamycin）、坦西莫斯、依维莫司及其它雷帕霉素衍生物，小分子抑制剂有Quinostatin、呋喃喹啉类、PI-103及其衍生物、NVP-BEZ235等^[26]^。

由于mTOR在肺类癌中表达升高，所以mTOR受体抑制剂可以使肺类癌细胞增殖活性降低，同时肿瘤血管生长因子的生成也减少。Moreno等^[27]^用雷帕霉素处理了类癌细胞系BON1以及NCI-H727之后，发现两个细胞系的增殖活性明显受抑制。而且雷帕霉素在抑制mTOR活性的同时也增加了mTOR上游AKT的磷酸化程度。Zatelli等^[28]^用依维莫司及SOM230处理了24例肺类癌患者的组织后，发现人嗜铬蛋白A（CgA）（20%）以及VEGF（15%）的分泌减少，p70S6K以及细胞的活力降低（30%），并且对依维莫司敏感的组织通常mTOR表达较高，相应患者血浆CgA的水平以及增值指数（ki-67）较高，但AKT的表达情况没有区别。另外，这两个实验均发现奥曲肽作为一种生长抑素受体抑制剂，单药使用可以抑制肿瘤细胞增殖，但是与mTOR受体抑制剂没有协同作用。

针对肺类癌细胞系，mTOR受体抑制剂显示出了极大的优势，这促使更多的科学家转而探索其在患者身上是不是也有很好的疗效。一项多中心参与的Ⅱ期临床研究^[29]^纳入了21例类癌、15例胰腺神经内分泌肿瘤患者，其主要治疗终点是评价患者接受坦西莫司的客观反映率。实验结果表明类癌组与胰腺神经内分泌肿瘤组的部分反应率类似，分别为4.8%和6.7%，总体中位疾病进展时间为6个月，1年的整体生存率为71.5%。并且坦西莫司可以显著的抑制p70S6K的表达（*P*=0.02）。在所有的患者中，p-mTOR基础水平越高，对坦西莫司的反应越好（*P*=0.01）。

依维莫司是种口服的雷帕霉素衍生物，2009年已先后被美国FDA和欧盟批准，用于治疗晚期肾癌。因为依维莫司在抗肿瘤细胞生长和增殖，抑制肿瘤血管生成方面的作用，所以其可以做为一种广泛的抗肿瘤药物，适用于包括神经内分泌肿瘤（包括肺类癌）、乳腺癌、肝癌、结节性硬化症在内的一系列肿瘤。一项Ⅱ期临床试验^[30]^通过研究不同RAD001药量联合奥曲肽30 mg在治疗18例类癌和13例胰岛细胞肿瘤中的疗效，发现患者的总反应率为15%，24周时的无进展生存率为71%，其中两个类癌患者获得部分缓解。最常见的副反应为轻微的口腔溃疡。G3/4期的副反应为贫血（1例）、血小板减少症（1例）、白血球减少症（1例）、疲劳（3例）、口疮性溃疡（2例）、腹泻（2例）、低血糖症（2例）、疼痛（2例）、皮疹（2例）、高血糖症（1例）、水肿（1例）、恶心（1例）。Yao等^[31]^分别用5 mg/d或者10 mg/d RAD001加上30 mg/d长效奥曲肽分析了30例类癌和30例胰岛细胞肿瘤患者，实验结果显示总体无进展生存期为60周，1年、2年以及3年生存率分别为83%、81%、78%。其中，在37例治疗前CgA水平升高的患者中，26例CgA水平在治疗后趋于正常或者有大于50%的降低。在治疗的第4周，患者LDH水平低于109 U/L的预后不良（*P*=0.01）。同上一实验类似，患者最常见的副反应为轻度的口腔溃疡，G3/4期毒副反应包括低磷酸盐血症（11%）、疲劳（11%）、腹泻（11%）。并且，依维莫司剂量在10 mg/d时，治疗效果明显优于5 mg/d。

RADIANT-2实验^[32]^研究了依维莫司联合长效奥曲肽在治疗包括类癌在内的低到中级神经内分泌肿瘤的疗效，共入组429例患者。最终结果显示依维莫司联合长效奥曲肽组的中位无进展生存期为16.4个月（95%CI: 13.7-21.2），安慰剂联合长效奥曲肽组为11.3个月。虽然此实验中肺类癌患者仅有44例，肺类癌亚群分析显示依维莫司组中位无进展期为13.6个月，明显优于安慰剂组的5.6个月（HR=0.72）。但是在治疗的副反应上，由于依维莫司组多以临床症状差、肺类癌和以前接受过化疗的患者为主，没有表现出明显的优势。其中，患口腔炎的人数为133（占总人数的62%）、59例腹泻（27%）、80例皮疹（37%）、67例疲劳（31%），而且3级-4级不良反应人数分别在14例（7%）、13例（6%）、2例（1%）、14例（7%）。与安慰剂组相比，副反应率分别为口腔炎（62%-14%）、腹泻（27%-16%）、皮疹（37%-12%）、和疲劳（31%-23%）。

在所有有关依维莫司在治疗肺类癌的实验中，患者均获得较长时间的缓解。并且基础CgA、NSE水平高的患者对依维莫司反应更好，治疗后患者血循环中CgA、NSE明显下降者疾病无进展生存期延长，预后好^[33]^。

## 4 结语

随着人们对发病率不高的肺类癌的研究进展，针对不同基因的靶向研究可以作为临床用药的强大支柱。对于PI3K/AKT/mTOR信号路径异常活化的患者，希望我们以后不仅可以做到抑制肿瘤生长，还可以实现恢复对某基因耐药患者的敏感性，使他们重新获益。相信今后肺类癌能够被更早更准确的诊断及治疗，患者能够获得更长的生存期，生活质量会更高。
